# Comparative Study on Microstructural Stability of Pre-annealed Electrodeposited Nanocrystalline Nickel During Pack Rolling

**DOI:** 10.1186/s11671-018-2749-1

**Published:** 2018-10-24

**Authors:** Haitao Ni, Haiyang Lv, Zhaodong Wang, Jiang Zhu, Xiyan Zhang

**Affiliations:** 1 College of Materials and Chemical Engineering, Chongqing University of Arts and Sciences, Chongqing, 402160 China; 2 College of Materials Science and Engineering, Chongqing University, Chongqing, 400044 China

**Keywords:** Nanocrystalline nickel, Pack rolling, Microstructural stability, X-ray diffraction analysis, Transmission electron microscopy

## Abstract

Microstructural stability is an important issue for nanocrystalline materials to be practically used in many fields. The present work shows how microstructure evolves with rolling strain in pre-annealed electrodeposited nanocrystalline nickel containing an initial strong fiber texture, on the basis of X-ray diffraction line profile analysis as well as transmission electron microscopy observation. The influence of shear strain on microstructural stability of the metal/roll contact interface is compared with that of the metal/metal contact interface; the latter would be closer to deformation in plane strain compression. From the statistical microstructural information, together with experimentally observed microstructure of deformed grains after the final rolling pass, it seems fair to conclude that the microstructure of the metal/metal contact interface is more stable during pack rolling than that of the metal/roll interface.

## Introduction

Nanocrystalline (nc) materials with grain size of less than 100 nm usually exhibit excellent mechanical properties especially high strength and high hardness that can be exploited in a wide variety of technological applications [1]. However, a large number of studies in recent years have shown that microstructural stability is an unavoidable and very important issue for nc materials when they are practically used [2–4]. As one of the most common microstructural features, grain size is always given first priority during the production and processing of nc materials. Unfortunately, it has been found that obvious grain growth may occur upon thermal treatment or mechanical processing of nc materials [5–9]. Following the classical Hall–Petch relationship for materials in a grain size range from ~ 20 to several hundreds of micrometers, grain growth will lead to performance degradation or deterioration. Throughout experimental investigations on relationship between microstructure and properties of nc metals, a large body of microstructural information was obtained by high-resolution transmission electron microscopy and/or three-dimensional atom probe [10–13]. These results seem to be direct and visible, but it is inevitably being questioned due to the fact that such local observation is too microcosmic. Therefore, it is necessary and important to understand the physics of plastic deformation from a more macro or an overall perspective.

Results from comparison of microstructure development in deformed nc materials have shown that grain growth behavior was influenced by initial microstructures such as grain orientation, internal stress, and crystal defect density [6, 14–16]. Thus, it is difficult to compare the results of microstructure evolution from other literature. Two or more samples are expected to deform simultaneously under nominally the same deformation condition. Note that accumulative roll bonding is one of the powerful techniques to produce ultra-fine grain microstructures by introducing large strain and strain gradient [17, 18]. Pack rolling has been chosen as the deformation processing route in our previous study. Effects of pack-rolling deformation on the microstructure, texture, and hardness of nc Ni have been primarily explored [19, 20]. It has been revealed that deformed zones with different grain sizes undergo different strains. But nonetheless, little attention is paid to in-depth comparative analysis of microstructural evolution such as the changes in crystal defect density. Therefore, the present study aims to further investigate the microstructural stability of pack-rolled nc Ni.

## Material and Methods

The fully dense electrodeposited nc nickel sheet with purity of 99.8% was selected as the present research materials. Prior to rolling deformation, the as-received sheet with thickness of ~ 0.22 mm was firstly annealed in vacuum at 373 K for 30 min to relief the residual stress. No evidence of obvious grain growth was found. Subsequently, the pre-annealed sheet was cut into small pieces with dimensions of 6 mm × 5 mm. Two pieces of samples with nominally similar initial microstructure, selected by X-ray diffraction (XRD) analysis, were stacked together and then went through a pair of rolls with a diameter of 180 mm at room temperature. After each rolling pass, it was found the two deformed samples had nearly the same thickness reduction. During such pack-rolling processes, the nominal rolling strain of each sample was determined by *ε* = $2\ln \left({t}_0/t\right)/\sqrt{3}$, where *t*_0_ and *t* are initial thickness and final thickness, respectively [21]. In this regard, we particularly focused on the microstructure evolution of the metal/metal contact interface and the metal/roll contact interface. For convenience, the metal/metal contact interface was referred to as interface M/M, and the metal/roll contact interface was referred to as interface M/R.

The deformation-induced microstructural changes were quantitatively examined by XRD analysis on a Rigaku D/MAX-2500 PC diffractometer with a rotary Cu target (18KW), operating in the fixed-time scan mode. Related microstructural parameters such as grain size and microstrain were obtained by X-ray diffraction line profile analysis [22, 23]. To verify the results obtained from XRD, transmission electron microscopy (TEM) was employed to make an intuitive evaluation on the final microstructure of normal direction-rolling direction section, particularly the grain size distribution. Foil samples for TEM were prepared by double-jet electropolishing in a solution of methanol and nitric acid (*V*:*V* = 4:1) at a temperature of 243 K. TEM observation was performed on ZEISS LIBRA 200FE at 200 kV of acceleration voltage. Grain morphology was observed in bright field imaging. Grain size measurements were conducted using dark field imaging accordingly. For each sample, more than 200 grains were measured to capture the overall evolution of grain size distribution. Furthermore, considering the limited dimension of small samples, microhardness measurement was conducted on both sides of the samples after each rolling pass, using HVS-1000 micro Vickers hardness tester with a load of 0.196 N.

## Results and Discussions

**Fig. 1:**
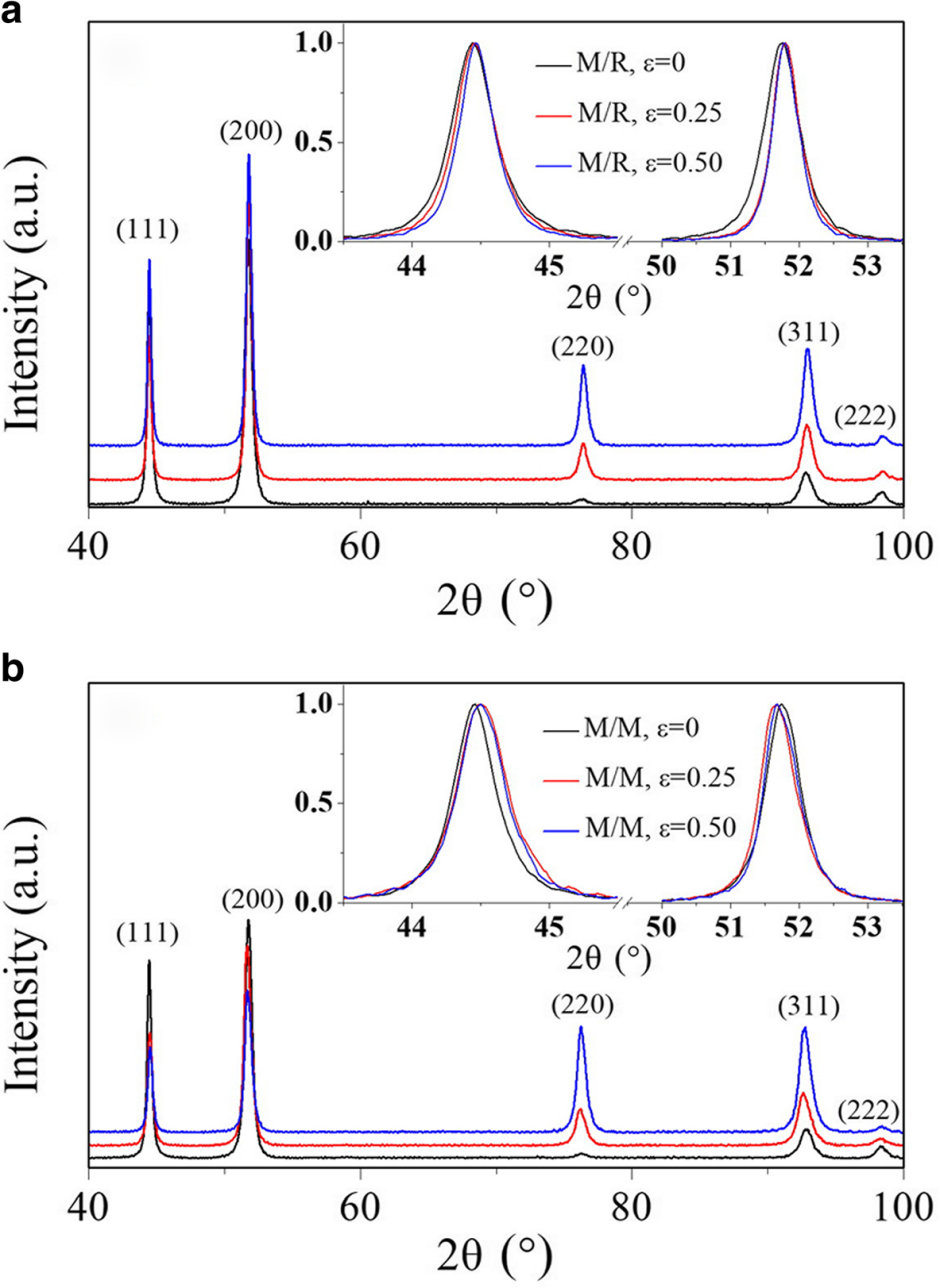
Typical XRD patterns of **a** the metal/roll (M/R) contact interface and **b** the metal/metal (M/M) contact interface during the pack-rolling deformation of pre-annealed electrodeposited nanocrystalline nickel. Normalized peaks of (111) and (200) reflection planes are displayed at the upper right corner

Figure 1 shows typical XRD patterns for the interfaces M/R and M/M of the pack-rolled nc Ni samples with different rolling strains. For the as-annealed undeformed samples (*ε* = 0), there is no remarkable difference in diffraction intensity between interface M/R and M/M. Further analysis on texture coefficient indicates the undeformed samples have an initial strong fiber texture. As expected, the diffraction intensities, especially for the (111) and (200) peaks, exhibit quite different texture evolutions after several passes of pack-rolling deformation (*ε* = 0.25 and *ε* = 0.50). According to the previous investigation involving deformation texture development, the interface M/R is dominated by shear deformation, while the interface M/M is closer to deformation in plane strain compression [24–26]. Quantitative analysis on the normalized results of the (111) and (200) peaks proves that there is a certain discrepancy between interface M/R and interface M/M. In the case of the interface M/R, the diffraction peaks are significantly narrowed, which is mainly due to the grain growth induced by deformation. However, in the case of the interface M/M, obvious peak broadening and peak shift are observed, indicating that a great deal of crystal defects such as dislocations and stacking faults have been produced during the rolling process.

**Fig. 2:**
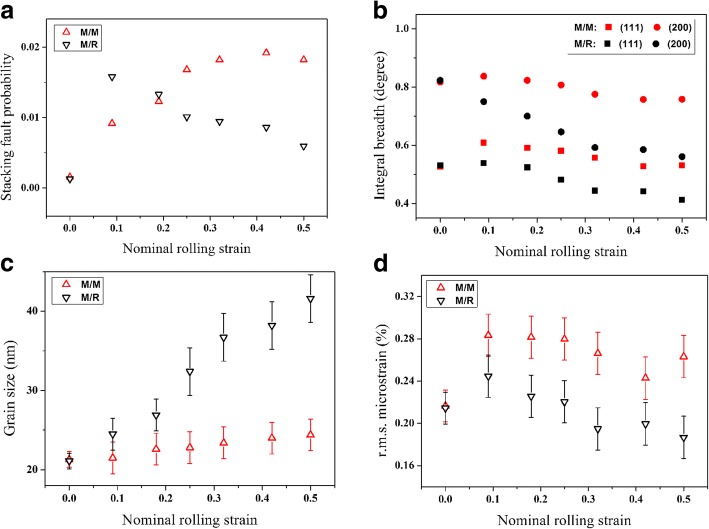
Quantitative results of **a** stacking fault probability, **b** integral breadths for the (111) and (200) peaks, **c** grain size, and **d** r.m.s. microstrain obtained by X-ray diffraction line profile analysis

Figure 2 shows the semi-quantitative results of nc Ni after each rolling pass, determined by the X-ray diffraction line profile analysis. The overall stacking fault probability (SFP), evaluated by peak shift, is shown in Fig. 2a. For the interface M/M, the overall SFP exhibits a relatively stable uptrend development with increasing strain. However, for the interface M/R, the SFP shows a sharp increase during the early stage of rolling deformation, reaching a maximum value of 0.015 at a small strain of ~ 0.1. Subsequently, this SFP turns to decrease with continuous deformation and get a value of 0.006 at a strain of 0.5, which is only one third as compared to the SFP of the interface M/M. Considered the generation mechanism of stacking faults in NC metals, such discrepancy indicates the microstructure of different interfaces should undergo different evolution routes.

Figure 2b shows the variation of integral breadths for the (111) and (200) peaks. It can be seen that the integral breadths of the two diffraction peaks of the interface M/M are significantly higher than that of the interface M/R during the whole pack-rolling deformation process. Particularly, it is noteworthy that there has been no large change in the integral breadth of the interface M/M, when comparing the final deformed state with the as-annealed state. In light of this, the evolutions of grain size and root-mean-square (r.m.s.) microstrain are carefully studied from the XRD line profile analysis. As can be seen in Fig. 2c, two interfaces of the deformed samples show a tendency of grain coarsening, but with different coarsening rates. The average size of grains located at the interface M/R increases more rapidly, which is proved by the following TEM observation. On the other hand, microstrain analysis indicates that there is a small increase in r.m.s. microstrain for both interfaces during the early stage of rolling deformation, as illustrated in Fig. 2d. With the continue of the deformation, the r.m.s. microstrain inside the interface M/R starts to steadily decline and reaches stability at a level of ~ 0.19%, while the r.m.s. microstrain inside the interface M/M tends towards stability at a level of ~ 0.26%. Such reduction in the r.m.s. microstrain is consistent with previous reports on the cold-rolled electrodeposited NC Ni-Fe alloy after large deformation. In combination with grain size evolution, the main reason for the decrease of r.m.s. microstrain would be associated to the grain coalescence and coarsening [27–29].

**Fig. 3:**
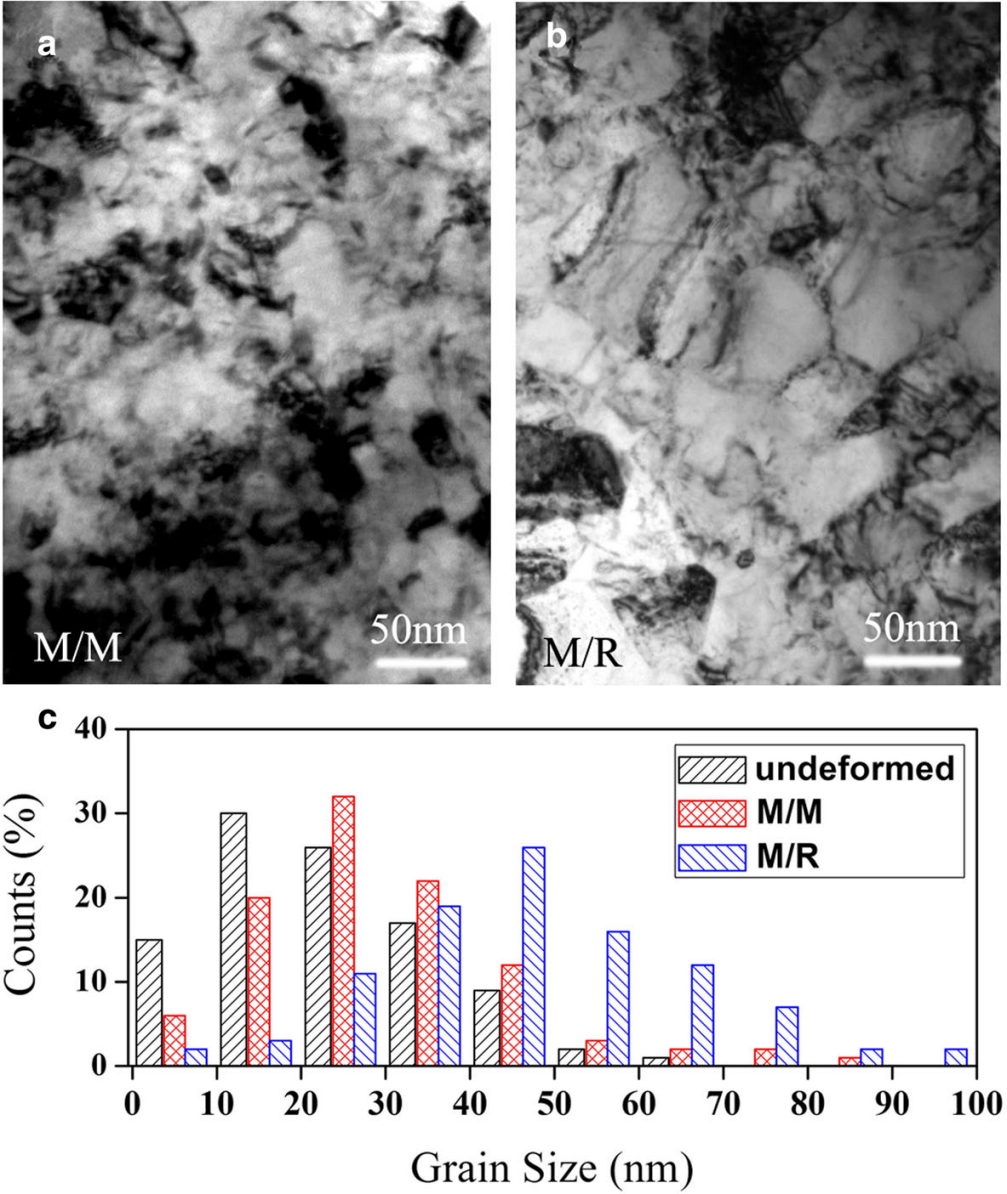
Typical TEM images of **a** the metal/metal (M/M) contact interface and **b** the metal/roll (M/R) contact interface after the final pack rolling pass. The grain size distribution before and after deformation is shown in **c**

Figure 3 shows typical TEM results of the interfaces M/M and M/R. It is clearly revealed that the grains located at the interface M/R is indeed larger than those located at the interface M/M after deformation. Further analysis on grain size distribution shows that a large proportion (more than 75%) of grains have a diameter less than 40 nm in the undeformed sample. After *ε* = 0.50 rolling deformation, the proportion of small grains (below 40 nm) drops obviously in the interface M/R. Instead, the proportion of large grains (above 50 nm) increases. Based on previous studies on dislocation activities in deformed grains, full dislocations would gradually start to dominate the deformation of large grains [30–33]. Thus, it is not difficult to understand the SFP of the interface M/M is much higher than that of the interface M/R.

**Fig. 4:**
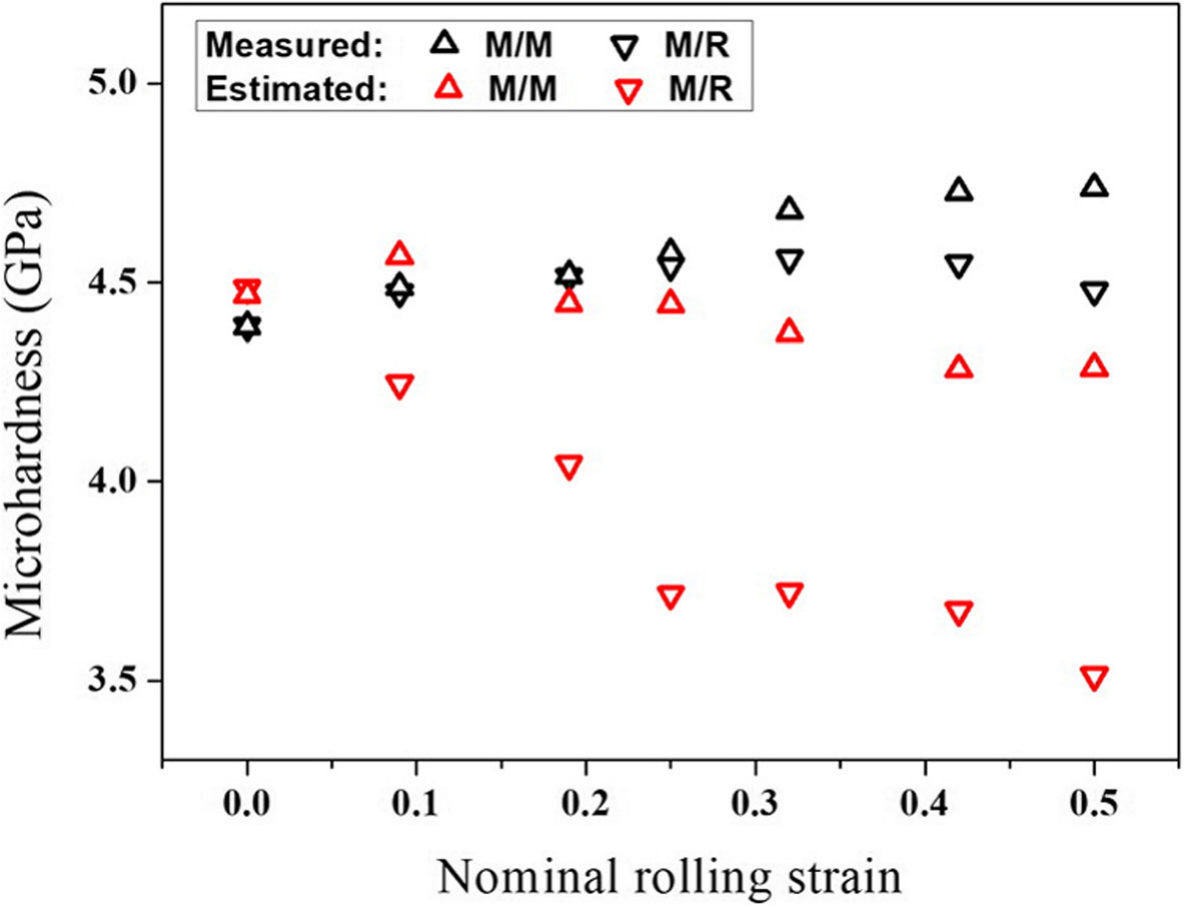
Experimental measurement and estimated prediction of microhardness evolution during the pack rolling of pre-annealed nanocrystalline nickel. The estimated values of microhardness are determined only by the grain size and dislocation density on the basis of the Hall–Petch relationship and the Bailey–Hirsch relationship

To correlate the microstructure evolution with mechanical response, microhardness variation of the interfaces M/M and M/R is shown in Fig. 4. There is no obvious disparity between the two interfaces at the early stage of deformation. As the strain increases, the microhardness of the interface M/M increases continuously, but the microhardness of the interface M/R appears to decline. On the other hand, comparing to the grain size and microhardness of samples in as-annealed state, deformation-induced strain hardening occurs at the interfaces M/M and M/R, despite the presence of grain coarsening. According to the classic Hall–Petch relationship, the microhardness will decrease with increasing grain size. Thereupon, for the as-deformed samples, the Bailey–Hirsch relationship is considered [34, 35]. The microhardness versus the square root of dislocation density is explored. It is no surprise to find a deviation from Bailey–Hirsch behavior. At the late stage of deformation, the remnant dislocation density, determined by the r.m.s. microstrain, is somewhat lower than the as-annealed state for the interface M/R, but the corresponding microhardness is somewhat higher. Herein, on the basis of the obtained microstructural information corresponding to a macroscopic area, it is a trial to explore the contributions of two common microstructural factors, namely dislocation density and grain size, to the microhardness. Taking the reported values or calculated values for nc Ni [36–38], the estimated values of microhardness are also displayed in Fig. 4. As a whole, the estimated values of the interface M/M is higher than that of the interface M/R, indicating indirectly that the statistical XRD results of microstructural evolution is credible. Furthermore, with comprehensive comparison and analysis on the gap between the estimated values and measured values, it is concluded that there should be another strengthening mechanisms inside the deformed nc samples, such as dislocation–dislocation interactions [37]. Especially for the interface M/R, dislocation–dislocation interactions could be present within large grains, helping enhance the degree of work hardening.

## Conclusion

In this work, the microstructural stability of nanocrystalline nickel during pack-rolling deformation was quantitatively investigated based on X-ray diffraction line profile analysis. The reliability of some relevant results was validated by transmission electron microscopy observation and microhardness measurement. The discrepancy in microstructural development between the metal/metal contact interface and the metal/roll contact interface was of particular concern. The results showed that the microstructures of the two interfaces underwent different evolution routes due to different imposed strains. From the statistical microstructural information such as crystal defect density and grain size, it can be concluded that the microstructure of the metal/metal contact interface exhibited more stable during pack rolling than that of the metal/roll interface.

## Abbreviations

M/M: Metal/metal
M/R: Metal/roll
nc: Nanocrystalline
r.m.s.: Root-mean-square
SFP: Stacking fault probability
TEM: Transmission electron microscopy
XRD: X-ray diffraction
